# Comparison of Two Pancreatic Anastomosis Techniques in terms of Postoperative Complications After Pancreaticoduodenectomy

**DOI:** 10.5152/eurasianjmed.2021.20194

**Published:** 2021-10

**Authors:** Suleyman Koc, Abuzer Dirican, Vural Soyer, Cengiz Ara, Saim Yologlu, Sezai Yilmaz

**Affiliations:** 1 Department of General Surgery, Cumhuriyet University School of Medicine, Sivas, Turkey; 2 Department of General Surgery, İnönü University School of Medicine, Malatya, Turkey; 3 Elbistan Government Hospital, Kahramanmaraş, Turkey; 4 Department of Biostatistics and Medical Information, İnönü University School of Medicine, Malatya, Turkey

**Keywords:** Pancreaticojejunostomy, pancreaticogastrostomy

## Abstract

**Objective:**

In this retrospective study, we compared the postoperative complications by using both the Clavien–Dindo classification and the Revised 2016 International Study Group on Pancreatic Surgery (ISGPS) classification methods after pancreaticoduodenectomy.

**Materials and Methods:**

The data of patients were retrospectively reviewed. Pancreaticojejunostomy (PJ) and pancreaticogastrostomy (PG) were performed on 41 and 40 patients, respectively. The patients were assigned into two groups for anastomosis types and compared with each other according to postoperative complications. The postoperative follow-up period of the patients was limited to 90 days.

**Results:**

No significant difference was detected between the two groups in terms of gender (*P* = .581) and age (*P* = .809). According to the Clavien–Dindo classification system, grade 1 complication rates were 29.3% and 35.0% in PJ and PG groups, respectively. Also, grade 2 complication rates were 34.1% and 32.5% in PJ and PG groups, respectively. Besides, grade 3B complication rates were 9.8% and 17.5% in PJ and PG groups, respectively. No grade 3A, grade 4A, and grade 4B complications were detected in both groups. But, grade 5 complications rates were 2.4% and 5.0% in PJ and PG groups, respectively. Based on the ISGPS classification system, the pancreatic fistulas were classified. The biochemical leak rates were calculated as 26.8% and 37.5% in PJ and PG groups, respectively. The rates were 14.6% and 10% in PJ and PG groups, respectively, for grade B complications. Also, grade C complication rates were 9.75% and 12.5% in PJ and PG groups, respectively. No statistically significant differences were detected between the two groups for postoperative complications.

**Conclusion:**

The evidence from this retrospective study suggests that there is no difference between the two types of pancreatic anastomosis techniques (PJ or PG) in terms of the rate of postoperative complications.

## Introduction

Pancreaticoduodenectomy (PD) is surgical resection of the pancreas and periampullary region. The process can be performed under different conditions, including malignancies, infections, and trauma.^1^ Pancreatic fistula, delayed gastric emptying, hemorrhage, acute renal failure, biliary fistula, intraperitoneal collection, peritonitis, and pleural effusion are common complications PD.^2^ The most frequently seen complication of PD is a pancreatic fistula. It is also the leading cause of postoperative morbidity and mortality.^3,4^ Different surgical techniques including simple closure of the pancreatic duct, pancreaticojejunostomy (PJ), pancreaticogastrostomy (PG), insertion of an external drainage catheter or stent into the pancreatic duct, anastomosis of the pancreas in the form of end-to-end invagination or end-to-side anastomosis of the jejunum, Roux-en-Y leg, two-layer or duct-to-mucosa anastomosis and total pancreatectomy are being used to reduce the incidence of the pancreatic fistula.^5^ Besides, drug therapies such as fibrinogen/thrombin-coated collagen patch are also used to reduce the incidence of these complications.^6^ However, the most preferred reconstruction techniques after PD are PJ and PG.^5^ Discordant results are being reported on the effect of these techniques to reduce the complications.^7^

As a standardized method, the Clavien–Dindo system was used for classifying surgical complications. The classification system is graded complications according to the type of therapy required to treat the complication.^8^ The Revised 2016 International Study Group on Pancreatic Surgery (ISGPS) classification and grading of postoperative pancreatic fistulas (POPFs) were used for classifying POPFs. When there is an increase in drain fluid amylase levels three times higher than the upper normal serum amylase level, the diagnosis of clinically relevant POPF is made.^9^ Although several studies have been performed on the evaluation of complications after PD, a limited number of studies evaluated the complications after PJ and PG in terms of both Clavien–Dindo and ISGPS classification and grading of POPF. Therefore, in this retrospective study, we compared the incidence of complications determined according to both classifications in patients on whom PJ or PG following PD was performed . We consider that this study contributes to the literature about PD complications.

## Materials and Methods

### Study Design

Between May 2009 and December 2018, a total of 81 patients aged between 21 and 75 years who underwent PD due to benign and malignant lesions of the pancreatic head, distal common bile duct, ampulla of Vater, duodenum and periampullary region, infection or trauma, were included in this study. Age, sex, preoperative albumin and bilirubin values, tumor localization, postoperative complications, treatment modalities of these complications, and length of hospital stay were recorded. Postoperative complications of the patients were classified according to the Clavien–Dindo and ISGPS classification and grading of POPF.^8^ The postoperative follow-up period of the patients was limited to 90 days. Ethics committee approval was received for this study from the Local Ethics Committee of Malatya İnönü University (Approval number: 2020/440). Because of the retrospective nature, we did not take informed consent from patients who participated in this study.

### Surgical Techniques

Reconstruction after pancreatic surgery remains under debate, and it is impossible to confidently conclude which method is better after PD. Therefore, the reconstruction method should be determined based on the patient and tumor characteristics, such as pancreatic duct diameter, consistency of pancreas, and oncological prognosis [1].

### PG Group

PD was performed a conventional resection, including the antrum. After removing the specimen, approximately 4–5 cm of the remaining pancreas was isolated from the retroperitoneum for PG reconstruction. A gastrotomy was performed to the posterior side of the antrum after insertion of a 6F or 8F feeding tube in the pancreatic duct; the pancreatic stump was placed as an invagination opening near the antrum in the posterior wall of the stomach. The other end of the feeding was removed from the anterior part of the stomach and fixed to the anterior gastric wall by tunneling with 3-0 Vicryl. The PG was constructed by following maneuvers: entire thickness polypropylene sutures (3/0) were passed from the posterior to the anterior pancreatic wall. Subsequently, the stitches were passed through the seromuscular layer of the posterior gastric wall. Then, entire thickness polypropylene sutures (3/0) were passed from the anterior to the posterior pancreatic wall. The duct of the pancreas was cannulated with a feeding tube, and this tube was fixed to the pancreas with polydioxanone sutures (5/0). During this procedure, the obstruction of the duct with the improper sutures was avoided. The posterior wall’s sutures were tied, and the pancreatic stump was inverted into the stomach. The stitches that had already been passed through the entire thickness of the pancreatic wall were passed through to the anterior seromuscular layer of the gastric wall, and the anterior anastomosis side was completed by tying these sutures. The anastomosis was checked with instilling the methylene blue via a nasogastric tube.^10^ On the other hand, in the PG group, biliary tract and enteral continuities were re-established with hepaticojejunostomy and gastrojejunostomy. Also, jejunojejunostomy was performed to prevent the bile reflux to the stomach.

### PJ Group

First, entire thickness polypropylene sutures (3/0) were passed from the posterior to the anterior pancreatic wall. Subsequently, the stitches were passed through the seromuscular layer of the isolated jejunum, which was previously prepared for anastomosis. The dorsal anastomosis side was completed by tying these sutures. An incision to the antimesenteric side of the jejunum equal to the pancreatic duct was created. Then, duct to jejunal anastomosis was performed with polydioxanone (5/0) interrupted sutures. A feeding tube was inserted and fixed to the pancreas with polydioxanone sutures (5/0) for all cases. Then, entire thickness polypropylene sutures (3/0) were passed from the anterior to the posterior pancreatic wall. The sutures that had already been passed through the entire thickness of the pancreatic wall were passed through to the anterior seromuscular layer of the jejunal wall, and the anterior anastomosis side was completed by tying these sutures.

After pancreas anastomosis, an end-to-side hepaticojejunal reconstruction on the first jejunal loop (50–60 cm from the Trietz) was performed, re-establishing the biliary tract continuity. After constructing the anastomosis between the stomach and the jejunum, the procedure was terminated in the group PJ.

Following hemostasis, 2 or 3 drains were placed around anastomosis. A nasogastric tube was used routinely for decompression purposes in all patients and removed when the output decreased below 300–500 cc daily on 3–5 postoperative days.

### Postoperative Care, Follow-Up, and Data Collection

All patients were followed up for at least 3 days in the intensive care unit for postoperative bleeding complications, especially in the early period. Prophylactic antibiotic administration was started in the perioperative period and was usually discontinued 3 days after surgery. Somatostatin infusion was started postoperatively and continued for 7 days. Routine total parenteral nutrition support was provided for all patients on the day after surgery and was discontinued once the patient tolerated the oral intake. Complete blood count, international normalized ratio, and biochemical parameters were studied daily in the first week. Daily output and characteristics of the drains (cirrhosis, sero-hemorrhagic, hemorrhagic, chylous, etc.) were recorded. Amylase from drain output was studied at least twice between 1 and 5 postoperative days. In case of suspicion of a pancreatic fistula or anastomosis leakage at postoperative follow-up, the patient underwent abdominal ultrasonography or abdominal computational tomography, and the results were evaluated by interventional radiology, gastroenterology, and infectious diseases specialists.

### Statistical Analysis

The D’Agostino & Pearson, q–q graphs, and histogram were used to assess the data’s conformity to normal distribution. The Mann–Whitney *U*-test and Student’s *t*-test were performed for total bilirubin, albumin for between-group comparisons. For comparing the differences between certain characteristics, the Chi-square was utilized. The analysis of the data was performed with SPSS for Windows version 22.0 software (IBM SPSS Corp.; Armonk, NY, USA). A value of *P* < .05 was considered statistically significant.

## Results

We included 81 patients in this study. Patients were grouped according to pancreatic anastomosis type as PJ and PG. PG anastomosis was performed in 40 patients, and PJ anastomosis was performed in 41 patients. In the PJ group, 29 patients were male, 12 were female, and the mean age was 62 years. In the PG group, 26 patients were male, 14 were female, and the mean age was 62 years. There was no significant difference between the two groups in terms of age (*P* = .809) and gender (*P* = .581) distribution. Among 81 patients, 1 patient had duodenitis due to cytomegalovirus, 1 patient had a pancreatic laceration, and 79 patients had malignancy. In one of our patients, a fibrous tissue developed in the duodenum secondary to cytomegalovirus infection caused serious stenosis in the biliary tract and Wirsung, and endoscopic interventions could not pass this stenosis. In the radiological imaging methods, tumoral images were obtained. Pancreatic laceration in our other patient, who underwent a non-malignant Whipple procedure, developed due to a traffic accident. The role of PD in trauma was best summarised by Walt ^11^. “Finally, to Whipple or not to Whipple, that is the question”. In the massively destructive lesions involving the pancreas, duodenum, and common bile duct, the decision to do a PD is unavoidable. Our patient had a laceration in a large area, including the pancreatic head, distal common bile duct, and duodenum. Malignancy types of 79 patients were given in Table 1. The mean postoperative hospital stay was 18.2 ± 18.3 days in the PJ group, whereas it was 18.2 ± 18.3 days in the PG group (*P* = .066). The number of patients who underwent portal vein resection due to intraoperative tumor invasion was 1 and 5 in PG and PJ, respectively. Statistically, a significant difference was observed between PJ and PG groups in terms of preoperative total bilirubin levels (*P* = .028). There was no statistically significant difference between the PJ and PG groups in terms of postoperative serum albumin levels (*P* = .332). Different complications have been detected in 67 of 81 patients, according to Clavien–Dindo Classification. Grade 1 complication rates were 29.3% and 35.0%, grade 2 complication rates were 34.1%, and 32.5%, and grade 3B complication rates were 9.8% and 17.5% in PJ and PG groups, respectively. No grade 3A, grade 4A, and grade 4B complications were detected in both groups. Grade 5 complications rates were 2.4% and 5.0% in PJ and PG groups, respectively (Table 2). Based on the ISGPS classification and grading of the POPF system, the pancreatic fistulas were classified. The biochemical leak rates were 26.8% and 37.5% in PJ and PG groups, respectively. The rates were 14.6% and 10% in PJ and PG groups, respectively, for grade B complications. Also, grade C complication rates were 9.75% and 12.5% in PJ and PG groups, respectively (Table 3). In the PJ group, the cause of death was sepsis. On the other hand, two patients of the PG group were lost due to intra-abdominal bleeding and anastomotic leakage. There were no statistically significant differences observed between groups in terms of postoperative complications in both classification systems.

## Discussion

Several postoperative complications (biliary fistula, wound infection, pancreatic fistula, delayed gastric emptying, and mortality) have been reported after PD. Different surgical techniques are being used to decrease these complications. PJ and PG are the two most preferred methods among surgeons. Conflicting results have been reported on the comparison of complications between PJ and PG groups. In the present study, the number of patients with general condition disorders, including sub-febrile fever, delayed gastric emptying, inability to remove gas–stool, and wound infection was lower in the PJ group than the PG group. However, the difference was not statistically significant. Aroori et al^12^. reported no statistically significant difference between the PJ and PG groups in terms of wound infection.^12^ In a meta-analysis made by Perivoliotis et al^13^. ıt has been shown that no statistically significant difference between the two groups regarding wound infection.^13^ Bassi et al^14^. reported lower delayed gastric emptying incidence in PG than PJ.^14^ Accordingly, we believe that both techniques can be used interchangeably in terms of general condition disorders, including wound infection and delayed gastric emptying.

Postoperative low hemoglobin could be seen because of blood loss via the abdominal drain, digestive tract, or abdominal cavity after PD.^15^ Low hemoglobin levels cause different symptoms, including irregular heartbeat, headache, and shortness of breath.^16^ In our patients, low hemoglobin levels developed in the postoperative period were replaced with erythrocyte suspension prepared under aseptic conditions. In the current study, we found no statistically significant difference between the two groups regarding low hemoglobin complications. The rates of low hemoglobin were 31% and 32.5% in PJ and PG groups, respectively.

Pancreatic fistula is one of the most common complications of PD.^17^ Several previous studies compared the incidence of a POPF between PJ and PG. Discordant results have been reported about the incidence of pancreatic fistula after PG and PJ in these studies.^12–14,18^ In a research made by Bassi et al.^14^, Aroori et al.^12^, Yeo et al^17^., no differences have been reported in terms of the incidence of a pancreatic fistula between PJ and PG.^12,14,18^ However, previous studies also reported lower pancreatic fistula rate with PG instead of PJ.^19–22^

We did not find any statistical difference between PJ and PG groups in the pancreatic fistula in the present study. Therefore, we thought that both PJ and PG might be used regarding pancreatic fistula. Approximately 2350 patients have undergone liver transplantation in our clinic from 2002 till the end of 2018. Due to the experienced hepatopancreatobiliary surgeons performed these procedures, we think that this effectively achieved similar results in both methods. Small sample size and limiting the postoperative follow-up period to 3 months were the study’s limitations.

The evidence from this retrospective study suggests no difference between the two types of pancreatoenteric anastomosis techniques (PJ or PG) in terms of the rate of postoperative complications. Therefore, both methods can be used interchangeably when these complications are taken into account. Future large-scale studies are still required to clarify the issues of the safety of these techniques.

**Main Points**

- Although the most preferred reconstruction techniques after pancreaticoduodenectomy (PD) are pancreaticojejunostomy (PJ), pancreaticogastrostomy (PG), discordant results are being reported in terms of the incidence of complications between the two techniques.
- Incidence of complications after PJ and PG were evaluated according to Clavien–Dindo and the International Study Group on Pancreatic Surgery (ISGPS) classifications.
- No difference was found between PJ and PG in terms of the incidence of postoperative complications.
- Both methods can be used interchangeably when these complications are taken into account.

## Figures and Tables

**Table 1.** Distribution of Malignancy Types of Patients

Malignancy type	Number of patients (%)
Pancreas head adenocarcinoma	33 (41.7)
Ampullary adenocarcinoma	22 (27.7)
Distal choledoch adenocarcinoma	6 (7.6)
Duodenal adenocarcinoma	5 (6.3)
Ampullary neuroendocrine tumor	3 (3.8)
Ampullary signet ring cell carcinoma	2 (2.5)
Distal choledoch adenosquamous carcinoma	1 (1.3)
Duodenal diffuse large B cell lymphoma	1 (1.3)
Pancreatic undifferentiated carcinoma	1 (1.3)
Pancreas head intramucosal carcinoma	1 (1.3)
Pancreas head adeno squamous carcinoma	1 (1.3)
Pancreas head neuroendocrine tumor	1 (1.3)
Duodenal undifferentiated carcinoma	1 (1.3)
Pancreas serous microcystic adenoma	1 (1.3)
**Total No. of patients**	79 (100%)

**Table 2.** Treatment and the Number of Complications according to Anastomosis Types

Grades (No. of patients)	Complication Types	PJ group (No. of patients)	PG group (No. of patients)	*P*
Grade 1 (n = 26)	General condition disorder	12	14	.580
Grade 2 (n = 27)	Low hemoglobin	14	13	.875
Grade 3A	None	None	None	
Grade 3B (n = 11)	Pancreatic fistula	3	6	.271
	Wound evisceration	0	2	.528
Grade 4A	None	None	None	
Grade 4B	None	None	None	
Grade 5 (n = 3)	Death	2	1	.586

**Table 3.** According to the Revised 2016 ISGPS Classification and Grading of POPF the Number of Complications in Both PJ and PG Groups

Complication Types	PJ group (No. of patients)	PG group (No. of patients)	*P*
BL (NO POPF)	11	15	.433
Grade B (POPF)	6	4	.527
Grade C (POPF)	4	5	.739

BL, biochemical leak; ISGPS, International Study Group on Pancreatic Surgery; PJ, pancreaticojejunostomy; PG, pancreaticogastrostomy; POPF, postoperative pancreatic fistula
